# Alteration of sheep coat color pattern by disruption of *ASIP* gene via CRISPR Cas9

**DOI:** 10.1038/s41598-017-08636-0

**Published:** 2017-08-15

**Authors:** Xuemei Zhang, Wenrong Li, Chenxi Liu, Xinrong Peng, Jiapeng Lin, Sangang He, Xuejiao Li, Bing Han, Ning Zhang, Yangsheng Wu, Lei Chen, Liqin Wang, MaYila, Juncheng Huang, Mingjun Liu

**Affiliations:** 1 Key Laboratory of Genetics, Breeding and Reproduction of Grass-Feeding Livestock, Ministry of Agriculture(MOA), Key Laboratory of Animal Biotechnology of Xinjiang, Urumqi, Xinjiang 830026 China; 2 Institute of Animal Biotechnology, Xinjiang Academy of Animal Science, Urumqi, Xinjiang 830026 China

## Abstract

Coat color is an important characteristic and economic trait in domestic sheep. Aiming at alteration of Chinese merino sheep coat color by genome manipulation, we disrupted sheep agouti signaling protein gene by CRISPR/Cas9. A total of seven indels were identified in 5 of 6 born lambs. Each targeted lamb happened at least two kinds of modifications, and targeted lambs with multiple modifications displayed variety of coat color patterns. Three lambs with 4 bp deletion showed badgerface with black body coat color in two lambs, and brown coat color with light ventral pigmentation in another one. The black-white spotted color was observed in two lambs with 2 bp deletion. Further analysis unraveled that modifications happened in one or more than two copies of *ASIP* gene, and moreover, the additional spontaneous mutations of D_9_ and/or D_5_ preceding the targeting modification could also involve the formation of coat color patterns. Taken together, the entanglement of *ASIP* modifications by CRISPR/Cas9, spontaneous D_9_/D_5_ mutations, and *ASIP* gene duplications contributed to the variety of coat color patterns in targeted lambs.

## Introduction

Animal coat color as one of the major breed characteristics is an important production and economy trait. The native coat color pattern has been thought to be an adaptation to cope with the environment and biological factors^1–3^. Previous studies proposed that sheep coat color may have correlation with reproductivity, body weight and energy homeostasis. In fur and wool animals, coat color is of importance for economic value since it associates with farmer’s preference and consumer’s favor. The standard wild type sheep coat color is generally dark-bodied with a pale belly^4^. As a result of artificial selection, the unique coat color phenotype reached a high frequency in breeds, like white coat color in fine wool merino sheep, and showed autosomal dominant inheritance^5, 6^. Since the coat color is an important breed characteristic and production trait in domestic sheep, substantial investigations have been focused on maintenance or alteration of the coat color in sheep breeding, and elucidation of the mechanism of coat color formation and regulation.

Agouti signaling protein is an endogenous antagonist of melanocortin in varieties of vertebrate species^7^. It is highly conserved in mammals and functions as a competitive inhibitor to prevent α-MSH binding to melanocortin 1 receptor (MC1R), resulting in inhibition of MC1R signaling and eumelanogenesis^8, 9^. The effect of *ASIP* as negative agonist to MC1R is to promote pheomelanotic phenotype which characterized as red/blonde pigment in contrast to eumelanin featured by dark brown or black pigment^10^. Loss of function mutations prevent it from binding to MC1R and effectively darken the coat color. Previous studies documented that sheep dominant white phenotype correlated with variation of gene copy number and deregulated expression of *ASIP* gene. In some sheep breeds, the white coat color is attributed to a 190 kb tandem duplication of *ASIP* gene, for instance, the Australia merino sheep^11^. Structure analysis of *ASIP* gene in merino sheep evidenced that all white merinos had at least one duplicated *ASIP* allele, whereas all the recessive black Merinos contained only a single allele^11^. A non-synonymous nucleotide substitution of *ASIP* gene was identified to associate with white versus non-white coat color variation in Finnsheep^6^. Variations in *ASIP* gene have also been associated with white versus non-white color in Soay sheep^12^. The spontaneous deletions of *ASIP* gene in merino sheep had been well characterized previously. A 5 bp deletion of exon 2 which resulted in a frame shift followed by a premature stop at 63 amino acids was recognized as a recessive mutation for black coat color^13^. Likely, a 9 bp deletion in exon 2 resulted in a loss of tripeptide(SRL), which may affect the function of the *ASIP* transport leader sequence, was recognized as a causal mutation for sheep black color^11^.

To date, the knowledge of the role and mechanism of *ASIP* gene in determination of coat color pattern predominantly relys on spontaneous mutations in farm animals. Precise modification of *ASIP* gene by genome manipulation offers a powerful means to comprehensively recognize the function of *ASIP* gene and get insight into the mechanism of coat color formation and alteration. CRISPR/Cas9 gene editing system has been widely used to precise modification of animal genes since it was applied in 2013. CRISPR/Cas9-mediated NHEJ can be used to disrupt gene by targeted removal of start codon, frameshift, or generation of premature stop, which effectively generates loss-of-function knockout models^14^. In attempt to get colorful fine wool sheep and furtherly understand the role and molecular mechanisms of *ASIP* in formation and regulation of coat color, hereby we applied CRISPR/Cas9 gene editing approach to disrupt sheep *ASIP* gene under the guidance of sgRNA. Five targeted lambs with loss-of-function mutations exhibited dark (black or brown) or black/white doted coat color. Targeting analysis found that each targeted lamb had more than one types of modifications and showed similar or different coat colors. We speculated that the variation of coat color phenotypes in targeted sheep was likely attributable to comprehensive factors of Cas9-mediated modifications, duplications and other spontaneous mutations.

Our work is the first time to gain the coat color alteration in farm animals by disruption of *ASIP* gene via CRISPR/Cas9. It offers technical approaches to change coat color and provides evidence and animal models to study the function and mechanism of *ASIP* gene in formation of coat color pattern in farm animals.

## Results

### Design of sgRNA targeting sheep *ASIP* gene

**Figure 1:**
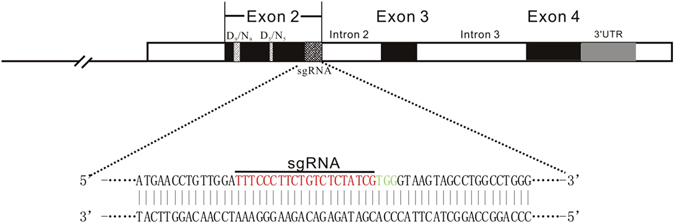
Schematic of the structure and diagram of sgRNA targeting exon 2 of sheep *ASIP* gene. Three coding exons and intervening intron sequences are showed in black and white boxes respectively. D_9_/N_9_ and D_5_/N_5_ are showed in heather gray, closed boxes. The sgRNA-targeting sequence is underlined and the PAM sequence is indicated in green.

To disrupt *ASIP* gene, we designed a sgRNA targeting exon 2 of *ASIP* gene, 137 bp downstream of translation start codon. The sequence of sgRNA was as below: TTTCCCTTCTGTCTCTATCGTGG. The PAM sequence was underlined (Fig. 1). Prediction and evaluation of sgRNA by online program showed highest level on the specificity and efficiency.

### Generation of *ASIP* gene targeted lambs

**Figure 2:**
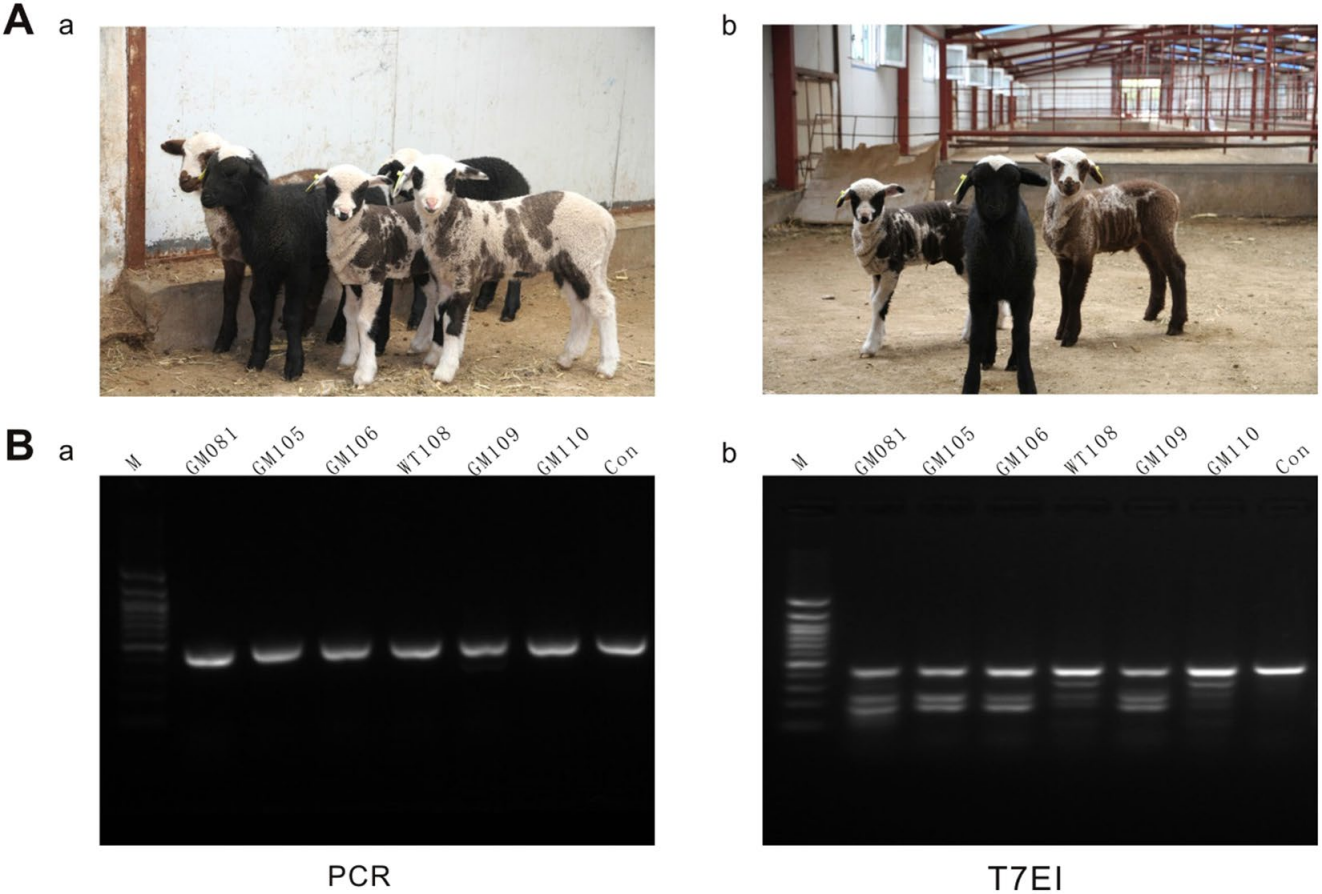
Coat color phenotypes of *ASIP* gene targeted lambs and primary detection of targeting modification. (**A)** Differential coat color patterns of *ASIP* gene targeted lambs. a, Photos of five lambs with *ASIP* gene targeted. b, Representative lambs of three distinctive coat color patterns. (**B**) Detection of sgRNA/Cas9 mediated modification by cleavage of *ASIP* gene with T7EI assay. a, PCR products of the targeting region of *ASIP* gene amplified by the DNA extracted from lamb tail tissues. b, Electrophoresis of T7EI cleavage assay of PCR products. M, 100 bp DNA ladder marker; Con, control of wild type lamb.

A total of 113 one-cell stage embryos were collected from 14 donor ewes, and 105 viable zygotes were subjected to cytoplasm microinjection of sgRNA/Cas9. All 92 cleaved embryos (92/105, 87.62%) were transferred to 60 surrogates, and after full-term gestation, 6 lambs were delivered. Interestingly, the coat colors in five lambs (tagged as GM081, GM106, GM110, GM109, GM105) displayed black, brown and white colorful coat, and only one (WT108) displayed white coat color (Fig. 2A-a and Table 1). The coat colors of five non-white lambs were characterized by three color patterns, black coat color with badger face in GM081 and GM106, brown/white dotted coat color with badger face in GM110, and black/white dotted body color with black-round eye in GM105 and GM109 (Fig. 2A-b).

**Table 1:** Summary on generation of CRISPR/Cas9 targeted lambs by injection of sgRNA/Cas9 mRNA into cytoplasm of zygotes.

Donor Ewe	Collected Embryos	Injected Embryo	Cleavage Ratio (%)	Embryos For ET	Surrogate Ewes	Conception Rate (%) (Pregnancies /Surrogates)	Lambs Born	Gene Modified Lambs	GM Ratio (%) (GM Lambs/ Lambs Born)
14	113	105	87.62 (92/105)	92	60	10 (6/60)	6	5	83.33 (5/6)

### Modifications and coat color phenotypes in targeted lambs

**Figure 3:**
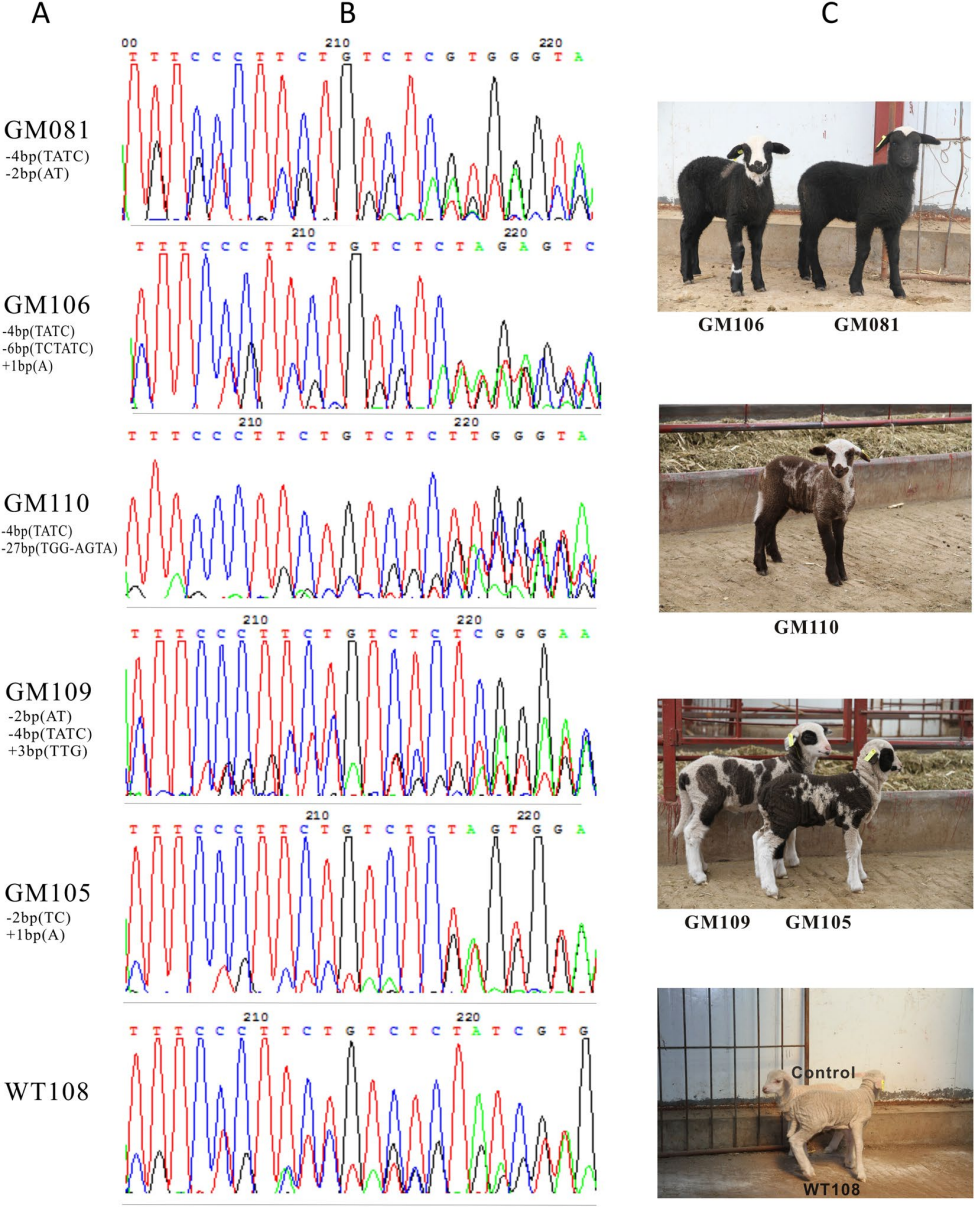
Distinctive coat color patterns and respective *ASIP* gene modifications of targeted lambs. (**A**) Targeted lambs with distinctive *ASIP* gene modifications. GM~ or WT~ numbers were the ear tags of each gene targeted (GM) or wild type (WT) lambs. “−” represents deletion, “+” represents insertion. (**B)** Photographs of sanger sequencing of each targeted lamb and non-targeted control. (**C)** Coat color patterns of each *ASIP* gene targeted lamb (GM081, GM106, GM110, GM109, GM105) and wild type or non-targeted control (WT108 and Control).

**Figure 4:**
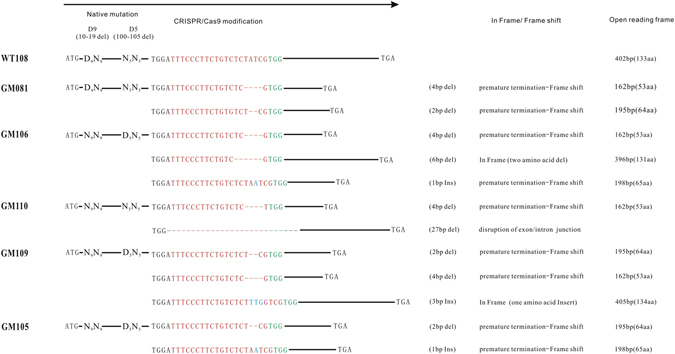
Diagram of the spontaneous deletions (D_9_D_5_ or N_9_N_5_) and sgRNA/Cas9 targeted modifications of five targeted lambs. “D_9_” was designated as spontaneous mutation (versus N_9_ as wild type), with 9 bp deletion downstream of ATG start codon. “D_5_” was designated as spontaneous mutation (versus N_5_ as wild type), with 5 bp deletion downstream of D_9_. The sgRNA/Cas9 targeted modifications were illustrated in the right of spontaneous mutation diagram, which showed the forms of modifications, events of in frame or frame shift in translation, and predicted protein products of premature stop or ablation of amino acids. The sgRNA sequence was marked in red and PAM sequence in green.

The skin tissues from tail were sampled and genomic DNAs were isolated from six lambs. All PCR products from 6 lambs were subjected to T7EI assay and sanger sequencing. Since the native mutations of D_9_ and D_5_ deletion in exon 2 of *ASIP* gene positioned extensively in prior to targeted loci, all the T7EI assay showed digestive profile (Fig. 2B), therefore the targeted events could only be determined by sanger sequencing. As expected, different types of targeted modifications were identified in 5 non-white coat color lambs, which were predominated by −4bp(~153–156,TATC) deletion, −2bp(~154–155/155–156,AT/TC) deletion, −6bp(~151–156,TCTATC) deletion, −27bp(~137–163, ATTTCCCTTCTGTCTCTATCGTGGGTA) deletion, and +1 bp(~154–155,A) insertion, +3 bp(~153–154,TTG) insertion, +17 bp(~153–154, TCCTCTGTTCCCTTCTG) insertion (Fig. 3A and B). For each targeted lamb, at least 2 forms of modifications raised by NHEJ were identified. Notably, even the lambs with similar dominant modifications, they displayed different coat color pattern (Fig. 3C). For instance, four lambs with 4 bp deletion (tagged as GM081, GM106, GM110, and GM109) appeared predominantly by black, brown, and black/white doted coat color. Lambs GM081 and GM106 with −4bp/−2bp and −4bp/−6bp deletions respectively both were black body with a white blaze on the head and neck in GM106, versus with white blade on the top head in GM081, whereas GM110 with −4bp/−27bp deletions showed dark brown coat with white blaze on the head, black lower legs, and white color on the hinder tail and neck bottom. The coat color of GM109 with −2bp/−4bp deletions which is the same modifications as GM081 appeared white/light-black doted body coat with black eye-round. The lamb GM105 with −2bp deletion and +1 bp insertion showed black-white doted body coat with black eye-round, which displayed the similar coat color to GM109. The only complete white coat color lamb was found without modifications, which was defined as wild type tagged by WT108. Further analysis revealed that the −4bp and −2bp deletions, and +1 bp insertion resulted in a frame shift and premature stop codon at 54, 65 and 66 amino acids respectively, and as consequence, led to a truncated ASIP protein. The 6 bp in-frame deletion was without frame shift and led to 2 amino acids (51 and 52 amino acids) ablation. However, the 27 bp deletions was happened spanning the exon/intron junction of exon2 and intron2, and presumably disrupted the splicing of ASIP transcription (Fig. 4).

### Genotyping of spontaneous deletions of D9 and D5 in *ASIP* gene targeted lambs

As the involvement of a spontaneous 9-bp deletion denoted as D_9_ (g.10–19 del/AGCCGCCTC) and a 5-bp deletion denoted as D_5_ (g.100–105 del/AGGAA) in exon 2 prior to sgRNA targeted loci, as well as the duplication of *ASIP* gene resulted from a tandem duplication of a 190-kb portion of the ovine genome at the *ASIP* locus^11^, it is difficult to determine the exact genotype of CRISPR/Cas9 modifications based on sequencing of PCR products only. The data of PCR product-based sequencing suggested that at least a copy of *ASIP* was homozygote with −4bp deletion in GM081, and likely, at least a copy of *ASIP* was homozygote with −2bp deletion in GM105. The genotype of targeted modifications required further evidence by T-A cloning sequencing. Previous investigations found that D_9_ and/or D_5_ spontaneous mutations were presented with high frequency in some sheep breeds^13^. Hereby we genotyped five targeted lambs and a wildtype (WT108) by D_9_ and D_5_ deletions (denoted D as mutation and N as wild type). As the result previously reported by Blinder *et al*., none of the white merinos were homozygous for D_9_ or D_5_, and either for both of the deletions^11^. All the six lambs (including a wild type) were heterozygous with only one mutation of D_9_ or D_5_. The genotype of N_9_N_9_ were identified in GM106, GM110, GM109 and GM105, and N_5_N_5_ were identified in GM081 and GM110 (Table 2). WT108 as wild type was D_9_N_9_ and N_5_N_5_ genotype, and with typical white coat in merino sheep. The genotypes of spontaneous mutations in five targeted lambs and wild type were shown in Fig. 4 and summarized in Table 2.

**Table 2:** Genotyping of spontaneous mutations, sgRNA/Cas9 targeted modifications and phenotypes of five targeted lambs.

Lambs	Targeted Modifications	Frame Shift/In Frame			Premature Stop	Native Mutation	Phenotype
GM081	−4bp(TATC)	3 N + 1	N = 1	trunkated	N53 Aa	D_9_N_9_N_5_N_5_	badger face with black body coat
	−2bp(AT)	3 N + 2	N = 0	trunkated	N64 Aa		
GM106	−4bp(TATC)	3 N + 1	N = 1	trunkated	N53Aa	N_9_N_9_D_5_N_5_	badger face with black body coat and white dot on the hinder back
	−6bp(TCTATC)	3 N	N = 2	deletion	/		
	+1 bp(A)	3 N + 1	N = 0	trunkated	N65Aa		
GM110	−4bp(TATC)	3 N + 1	N = 1	trunkated	N53 Aa	N_9_N_9_N_5_N_5_	Brown with badgerface and white color on the hinder tail and neck botom
	−27bp(TGG-AGTA)	3 N	N = 9	deletion	/		
GM109	−2bp(AT)	3 N + 2	N = 0	trunkated	N64 Aa	N_9_N_9_D_5_N_5_	Black/Brown-White dot color(with white on the head, leg and neck)
	−4bp(TATC)	3 N + 1	N = 1	trunkated	N53 Aa		
	+3 bp(TTG)	3 N	N = 1	deletion	/		
GM105	−2bp(TC)	3 N + 2	N = 0	trunkated	N64 Aa	N_9_N_9_D_5_N_5_	Black and white dotted
	+1 bp(A)	3 N + 1	N = 0	trunkated	N65 Aa		
WT108	/	/	/	/	Wild type	D_9_N_9_N_5_N_5_	white

Footnotes: N, denotes the number of triplet code; 3 N, denotes “in frame”; 3 N + 1 and 3 N + 2 denote “frameshift”.

### Verification of genotyping by T-A cloning sequencing

**Figure 5:**
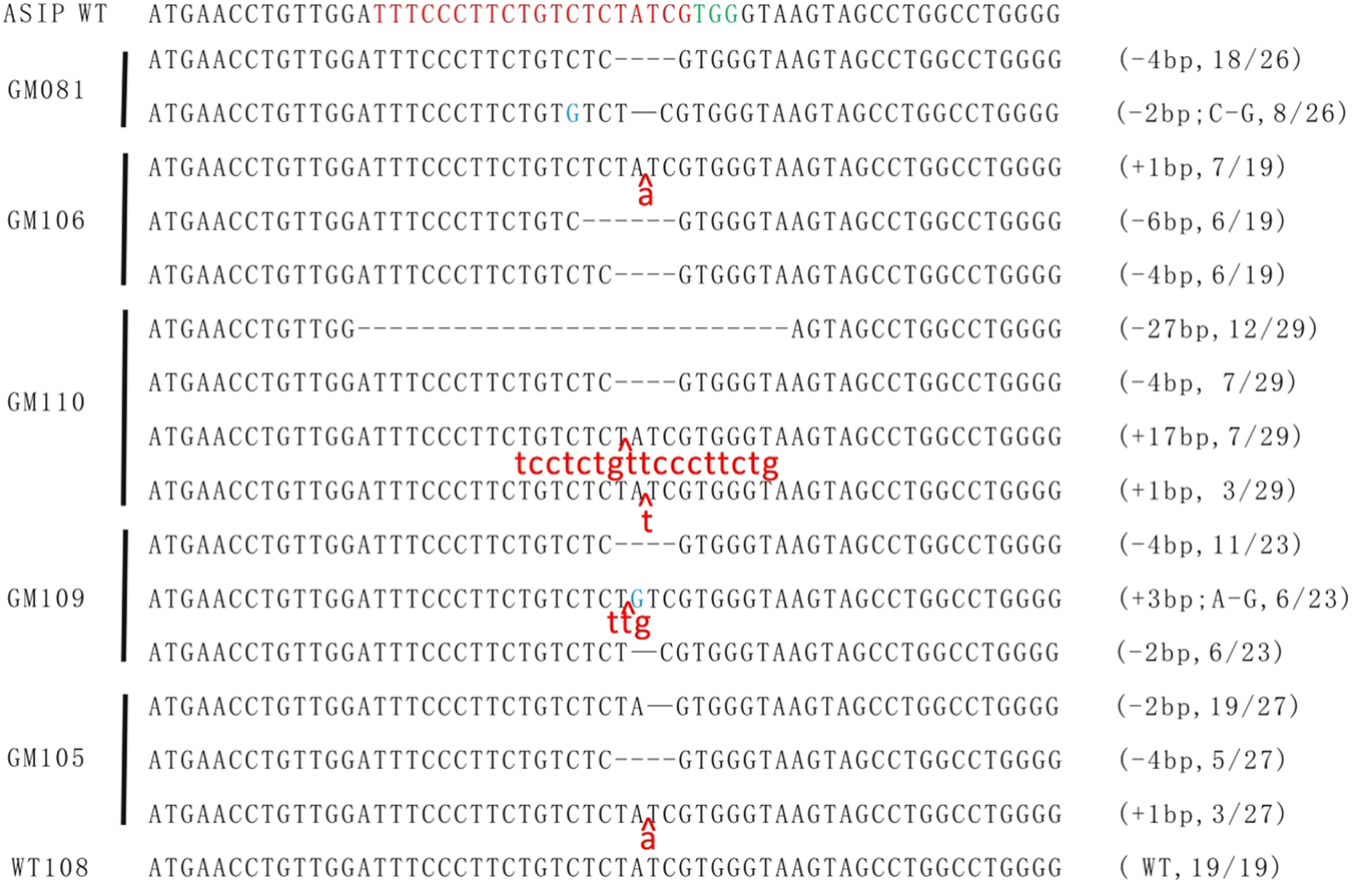
Varieties of targeted modifications of five targeted lambs. The left listed the targeted and wild type lambs. Each type of modifications was illustrated in the sequence in right of each lamb, which was determined by T-A cloning sequencing. The sgRNA sequence was marked in red and PAM was in green. The targeted modifications and ratios of colonies with modifications in the total colonies were displayed in the right. At least 19 T-A cloning colonies derived from the PCR products of each targeted lamb were sequenced. “−” represented deletion, “+” or “∧” represented insertion. N/N indicated the number of sequences with respective modification to the total sequencing colonies.

Since the existence of spontaneous mutations of D_9_ and D_5_ prior to Cas9 targeted loci and multiple copies of *ASIP* gene, genotyping of *ASIP* targeted sheep by CRISPR/Cas9 became much more complicated under such a context only by PCR-product based sequencing. Therefore we performed T-A cloning sequencing of colonies derived from each amplicon of five targeted lambs and a wild type. Based on the ratio of genotypes in total colonies, we testified the genotyping data derived from PCR-product based sequencing. All the genotypes of spontaneous mutations (D_9_/N_9_, D_5_/N_5_, N_9_N_9_ and N_5_N_5_) were in line with those from PCR-product based sequencing data (Table S1). Quite similarly, all the modifications identified by PCR-product based sequencing were found by colony-based sequencing as well. However, extra modifications which were not found in PCR-product based sequencing were identified in cloning sequencing, for instance, an extra +3 bp insertion and a A to G substitution in GM109 were not uncovered by PCR-product based sequencing, but were screened out from cloning sequencing Fig. 5). Two homozygotes with −4bp deletion in GM081 and −2bp deletion in GM105, which were suggested by PCR-product based sequencing, were also testified by colony-based sequencing, up to 70% (18/26) colonies with −4bp deletions in GM081 and 70% (19/27) colonies with −2bp deletions in GM105.This phenomenon inferred that multiple modifications in the same allele or different alleles could be happened in CRISPR/Cas9 mediated targeting.

### Association of coat color patterns with *ASIP* gene modification in targeted sheep

Sheep *ASIP* gene has been characterized as causative of the dominant white and tan allele and loss of function mutations of *ASIP* were associated with the black recessive non-agouti allele^11, 13^. Hereby we observed the similar phenomenon in *ASIP* gene disrupted sheep. Two lambs (GM081 and GM106) genotyped as −4bp/−2bp deletions and −4bp/−6bp/deletion plus +1 bp insertion respectively, which happened reading frame shift and premature stop codon in N-terminal of ASIP protein, exhibited black coat color with badger face(GM106) and a white blade on the top of the head (GM081). The GM110 with a −4bp and −27bp deletion led to a premature stop codon and a disruption of transcriptional splicing (−27bp deletion) respectively showed the brown/white dotted coat color with badger face. Though lambs of GM109 (with both −4bp and −2bp deletion and an extra in frame 3 bp insertion) and GM105 (with both −4bp and −2bp deletion and an extra +1 bp insertion) existed same premature stop codon, they displayed the different coat color pattern. GM109 was predominated by white coat with black dotted color, whereas the coat color of GM105 was predominated by black coat with white dotted color. Both lambs showed black-round eye. The coat color patterns indicated that similar modifications of targeted lambs displayed different coat color patterns. It inferred that the coincidence of targeted modification, spontaneous mutation and multiple copy of *ASIP* gene may entangled to affect the coat color pattern.

In summary, disruption of *ASIP* gene by CRISPR/Cas9 led to the alteration of coat color pattern in merino sheep and the variety of changed coat color patterns were most possibly associated with the entanglement of targeting modification, spontaneous mutation and multiple copy of *ASIP* gene.

## Discussion

In mammalian species, animal coat color is regulated by two main types of melanin: eumelanin and pheomelanin, which are derived from the amino acid tyrosine. The eumelanin is featured by dark brown/black pigment and the pheomelanin is characterized by red/blonde pigment. Tyrosinase is the rate-limiting enzyme to catalyze melanogenesis. When MC1R is activated and cAMP level is high, melanocytes produce more eumelanin, whereas when MC1R is inactivated and cAMP level is low, melanocytes produce more pheomelanin instead^15^. Sheep coat color is determined by the levels of eumelanin and pheomelanin in skin melanocytes as well. MC1R/cAMP signaling pathway plays critical role in the formation of coat color pattern.

**Figure 6:**
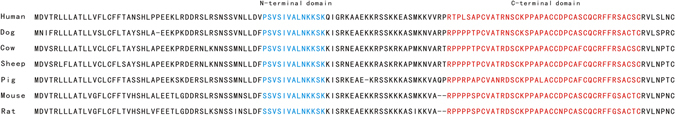
Amino acid sequences and primary ASIP protein structure of seven animal species. The alignment of amino acid sequences of seven animal species was listed. Amino acids highlighted in blue and red indicated regions of high local constraint that correspond to the N-terminal domain or C-terminal domain of ASIP protein respectively. Accession numbers for ASIP orthologs (and their species and sources) are P42127 (human, UniProt), Q5UK76 (dog, UniProt), Q29414 (cow, UniProt), A7UGE3(sheep, Uniprot), Q6ZYM3 (pig, UniProt), Q03288 (mouse, UniProt), and Q99JA2 (rat, Uniprot).

Agouti protein is a paracrine-signaling molecule which is normally expressed in the skin and with high homology among mammals. It functions as a competitive MC1R inhibitor to downregulate the synthesis of cAMP, and results in inhibition of eumelanogenesis, displays a pheomelanotic coat phenotype. Sheep ASIP has 133 amino acids (A_α_) in length, including 30 N-terminal (39–68 A_α_) and 34 C-terminal (94–128 A_α_) residue domains (Fig. 6). ASIP functions depending on one of a major accessory protein: attractin, a large single-transmembrane-spanning domain protein that is required for *ASIP* signaling *in vivo*, and is thought to act as an accessory receptor for *ASIP*-mediated antagonism of MC1R^7^. The N-terminal domain of *ASIP* is required for interaction with attractin, therefore the N-terminal is essential for interaction with MC1R. The C-terminal of *ASIP* is sufficient for potent antagonist function at their cognate melanocortin receptors. Since both the N-terminal and C-terminal domains are necessary for *ASIP* function, any loss-of-function mutations of *ASIP* gene could perturbate the MC1R signaling pathway and interfere the melanogenesis.

As a result of artificial selection, the white coat phenotype has reached a high frequency and shows autosomal dominant inheritance in Merino sheep. Investigations on the genetic basis of merino sheep coat color uncovered that the dominant white color has close association with *ASIP* gene structure and function. Loss-of-function mutations of *ASIP* could result in dark brown or black coat color^11^.

In attempt to generate colorful fine wool sheep and furtherly understand the role and molecular mechanisms of *ASIP* gene in formation and regulation of coat color, *ASIP* gene was targeted by CRISPR/Cas9. All five none-white lambs had distinctive indels at targeted loci. Analysis of the editing event by sequencing found that most of predominant modifications, like −4bp and −2bp deletions, and +1 bp insertion, happened premature stop at 54, 65, and 66 amino acids in N-terminal domain respectively, and resulted in frame shift and premature termination. These premature terminations all happened in N-terminal and consequently led to ablate the complete C-terminal domain. Even the −6bp in frame mutation caused deletion of two amino acids (51 and 52 amino acids) at N-terminal may interfere the function of N-terminal domain.

On the base of data from PCR-product based sequencing (Fig. 3B and Table 2), we observed that each targeted lamb happened multiple editing events in one or more alleles of *ASIP* gene and showed similar or different coat color. GM081, GM109, and GM105 had the common modifications of −4bp and −2bp deletions. However, the coat color patterns were apparently different. Lamb GM081 showed whole body black with white blade on the top of the head, whereas the GM109 displayed white body with black-dotted coat, and GM105 displayed black body with white-dotted coat color pattern. Further analysis of the ratio of each modification depending on cloning sequencing revealed that GM081 was dominated by −4bp deletion (with 70% colonies), thus we deduced that it could be a homozygote of −4bp deletion in at least one copy of *ASIP* gene. In contrast, the colonies with −4bp deletion in GM105 was only 18.5% (5/27), while those with −2bp deletions accounted up to 70% (19/27) (Fig. 5), therefore the GM105 could have at least a homozygous alleles with −2bp deletion. Besides multiple modifications, duplication of targeting gene made the editing events and effects more complicated. Depending on previous investigations, all white merinos had more than one duplicate *ASIP* alleles, while all the recessive black merinos contained only single copy allele^11^. Regarding to NHEJ feature resulted from CRISPR/Cas9, the modifications could happen in any targeting alleles randomly, and each allele may have different modifications. As consequence, the modifications led by CRISPR/Cas9 in multiple copy genes hold the polymorphic character. Due to the technical constrains of PCR-product based sequencing, some of modifications could not be observed from PCR-product based sequencing, so sequencing more colonies of PCR product was necessary to find more modifications for multiple copy gene. Given that editing and analysis of targeted multiple copy gene could be much more complicated than that of single copy gene, the exact editing events, genotypes and targeting effects should be taken into serious considerations. More importantly, the precise targeting approaches on multiple copy genes are worthwhile to develop in the future.

Owing to the multiple copy and spontaneous mutations of D_9_ and D_5_ of *ASIP* gene in merino sheep, we could not determine which and how many copies happened modifications, and exactly differentiate the homozygote and heterozygote. It could be the reason why different appearance of coat color pattern displayed in lambs with similar modifications. Nonetheless, despite the complicated *ASIP* gene context, our results revealed that sheep *ASIP* gene plays critical role in formation of sheep coat color, and modifying the *ASIP* gene can alter the sheep coat color. Our work shows great potentials to gain favorable coat color by editing *ASIP* gene and fosters animal model to aid future study on the mechanisms of formation and regulation of sheep coat color pattern by *ASIP* gene.

By optimizing donor treatment and embryo micromanipulation (including the superovulation protocol, the time of mating and embryo harvesting, and the time of embryo microinjection and transfer), high efficient one-cell embryo collection and gene modification were achieved. However, the conception of recipients was very low, with only 10% (6/60) comparing to over 40% conception of our early result in regular embryo transfer. Investigations on human and animal model elucidated a role of the melanocortin pathway in immunity, energy homeostasis, and reproduction^16, 17^. However, extensive functions have been addressed in sheep *ASIP* gene except pigmentation so far. Hereby we hypothesize that the low pregnancy perhaps is owing to the other functions of *ASIP* which have not been recognized and may affect the embryo development or fetus survive. Further investigations on the extensive functions of *ASIP* are needed to be carried out in the future.

Taken together, alteration of coat color in targeted sheep versus wildtype was observed by disruption of *ASIP* gene in merino sheep with CRISPR/cas9. The variety of coat color patterns could be attributed to diverse modifications combined the spontaneous mutations and duplication of *ASIP* gene. Our work demonstrated that sheep *ASIP* gene plays critical role in determination of coat color, and the coat color can be changed by disruption of *ASIP* gene. The targeted lambs with variety of coat color by gene editing can be as a model to study the mechanisms of *ASIP* gene function. Furtherly, the complicated context of *ASIP* gene in targeted lamb also provides a proper model to study the gene editing events and technical approaches in multiple copy genes.

## Methods

### Ethical statement

The animals used in this study were regularly maintained in the Research Base of Sheep Breeding of Xinjiang Academy of Animal Science. Surgeries were performed under strict aseptic conditions, and efforts were made to minimize animal suffering. All animal handling procedures were carried out in strict accordance with approved guidelines of the Institutional Animal Care of Xinjiang Academy of Animal Science(Approval ID: 2016ZX08010-004-009).

### sgRNA design

The sgRNA targeting sheep *ASIP* gene was designed by the online program (http://www.e-crisp.org/E-CRISP/reannotate_crispr.html) based on 169 bp exon2 sequence (GenBank: NM_001134303.1). The schematic structure of sheep *ASIP* gene and diagram of sgRNA design was showed in Fig. 1A. The off-target effect was predicted in Ovis aries Oar_v3.1 genome data by the program.

### Preparation of Cas9 mRNA and sgRNA

Corresponding oligos were ordered from ShangHai Sangon Biotech Corporation. Paired synthesized oligonucleotides (*ASIP*-sgRNA-CF/CR, Table S2) for sgRNA preparation were annealed and phosphorylated as follows. Initially, 1 μl of sense oligos, 1 μl of anti-sense oligos, 2 μl of 10× annealing buffer, 2 µl of ATP (10 mM), 1 µl of T4 Polynucleotide Kinase, and 13 µl of ddH_2_O were mixed at 37 °C for 30 min, incubated at 95 °C for 5 min, and cooled at room temperature. Meanwhile, the bicistronic expression vector pX330 (Addgene, Cambridge, MA, USA), which was used to express Cas9 and sgRNA, was linearized by *BbsI*, treated with CIP(Alkaline calf intestinal phosphatase, NEB, Beverly, MA, USA), and gel purified. Then the phosphorylated oligonucleotide was subcloned into linearized pX330 vector containing two expression cassettes, a human *Streptococcus pyogenes* (*hSpy*) Cas9 and the chimeric gRNA, thereby constituting a recombinant vector for the preparation of Cas9 and sgRNA by *in vitro* transcription.

For the production of sgRNA by *in vitro* transcription, a T7 promoter sequence (TAATACGACTCACTATAGG) was added upstream of the sgRNA, and at the 5′end of sgRNA sequence, two G nucleotides were appended for optimal transcription. Primers used in preparation of template for *in vitro* transcription were listed in Table S2 (*ASIP*-sgRNA-TF/TR). The PCR reaction included 25 µl of PrimeSTAR Max Premix, 0.5 µl of each T7-sgRNA oligos (10 µM), 50ng of the recombinant vector, and ddH_2_O to a final volume of 50 µl. The mixture was then run in a thermocycler under the conditions at 95 °C for 5 min; 35 cycles of 98 °C for 10 s, 55 °C for 15 s, 72 °C for 5 s; and final at 72 °C for 10 min. The amplified T7-sgRNA product was subjected to gel purification(Qiagen, Frankfurt, Hessen,Germany) and used as the template for *in vitro* transcription by a MEGAshortscript^TM^ T7 transcription kit (Ambion, Austin, Texas, USA).

A three-nucleotide spacer (AGA) and T7 promoter were added to the 5′ flanking of Cas9 coding region by PCR with the primers of Cas9 F/R (Table S2), and with px330 as the template. The amplified T7-Cas9 PCR product was gel purified and used as the template for *in vitro* transcription with a mMESSAGE mMACHINE® T7 ULTRA transcription kit (Ambion, USA). After transcription, a poly(A) tail addition and DNase I treatment was performed in accord to the manufacturer’s instructions for Cas9-encoding mRNA.

Both the Cas9 mRNA and the sgRNA were purified using a MEGAclear^TM^ transcription clean-up kit (Ambion,USA), eluted into RNase-free water, and frozen at −80 °C. A Nanodrop spectrophotometer was used to ensure purity and measure concentrations. The qualities of the RNAs were further checked by gel electrophoresis.

### Generation of gene-modified sheep by injection of Cas9/sgRNA into zygote

Gene-modified sheep were generated via microinjecting the mixture of 100 ng/µL Cas9 mRNA and 50 ng/µL sgRNA into the cytoplasm of zygotes. Sheep zygotes were collected through surgical oviduct flushing from the donors by estrus synchronization and superovulation treatment as described with slight modifications^18, 19^. In brief, donors were treated with CIDR (progesterone 300 mg) maintaining in the vagina for 12 days. Superovulatory treatment was carried out by administration of 240 mg oFSH (ovine follicle stimulating hormone, sansheng) in six decreasing i.m. doses every 12 h (50 mg × 2, 40 mg × 2 and 30 mg × 2) starting 3 d after estrus detection (Day 0). Removal of the plug and injection of a single dose i.m. of 0.1 mg cloprostenol (sansheng) was carried out when completing the fifth injection of FSH. After plog removal, ewes were tested to determine the onset of estrus behavior by twice-daily visual observation (8:00 AM and 8:00 PM) with introduction of a vasectomised ram for 30 min. If the ewes were in oestrus, injection of 200 mg LH (sansheng) was performed.

Sheep zygotes (around 20 h post-fertilization) were surgically collected and immediately transferred into SOF medium with 3 mg/mL BSA. Then the zygotes were subjected to cytoplasmic microinjection with the mixture of Cas9/sgRNA. After injection, the zygotes were cultured in *in vitro* culture medium [SOF supplemented with 3 mg/mL bovine serum albumin (BSA)] for 24 h at 38.6 °C, 5% CO_2_ until they divided into 2~4 cells.

68 ewes at the age of 2~4 years old with regular estrus cycles were selected as recipients. For embryo transfer, recipients were synchronized by the same treatment as donor ewes. The divisive embryos were transferred into the ampullary-isthmic junction of the oviduct of recipients. After 60d transplantation, pregnancy was determined by the ultrasound scan.

### PCR-based assay

For editing event analysis, tissue of lamb tail was sampled and the genomic DNA was extracted from ground tissue lysate by phenol-chloroform, and recovered by alcohol precipitation. The DNA with equal amount (300ng) was used as template for PCR amplification by primers (*ASIP*- F/R, Table S2) according to the following program: 95 °C for 5 min followed by 95 °C for 30 s, 61 °C for 30 s, 72 °C 45 s,, and ended by 72 °C for 7 min after 35 cycles.

### T7EI cleavage assay

The T7 endonuclease I (T7EI) cleavage assay was performed as described^20^. In brief, targeted fragments were amplified from extracted DNA by Taq DNA polymerase (BBI, Toronto, Canada). Around 200 ng PCR product for each sample was denatured and reannealed to generate heteroduplexes in NEB Buffer 2(NEB, Beverly, MA, USA) using the following program setting: 95 °C for 10 min, 95 °C–85 °C at −2 °C/s drop, followed by 85 °C–25 °C at −0.3 °C/s drop, hold at 25 °C for 1 min, and finally at 10 °C. After reannealing, the products were treated with 0.2 µl T7EI (NEB, USA) and incubated at 37 °C for 30 min. Then, the digested PCR product was subjected to 2% agarose gel electrophoresis.

### DNA sequencing of targeted gene

For verification of indel mutations, PCR products were sequenced directly (primers used were listed in Table S2) to testify the presence of editing events. Those with overlapping peaks were then sub-cloned into pMD-19T vector (TakaRa, Japan) for sequencing. For each sample, at least 20 colonies were randomly picked out and sequenced using M13 primer. Mutations were identified by alignment of the sequenced alleles to the wild type.

## Electronic supplementary material

Supplementary Information
