# Supplementary material for: Alteration of sheep coat color pattern by disruption of ASIP gene via CRISPR Cas9
Source: Sci Rep. 2017 Aug 15;7:8149. doi: 10.1038/s41598-017-08636-0 (PMC5557758; doi:10.1038/s41598-017-08636-0)
Supplement: Supplementary file 1 — Supplementary Information [file 41598_2017_8636_MOESM1_ESM.pdf]

Table S1. Genotyping of spontaneous deletion of D9 and D5 by T-A cloning sequencing of 5 targeted lambs.

Lambs	Total Number of Independent Colonies Been Sequenced	Ratio of D_9 or N_9 Colonies in Total Colonies		Ratio of D_5 or N_5 Colonies in Total Colonies		Genotype
		D_9	N_9	D_5	N_5	
GM081	26	7/26	19/26	/	26/26	D_9N_9/N_5N_5
GM106	19	/	19/19	6/19	13/19	N_9N_9/D_5N_5
GM110	29	/	29/29	/	29/29	N_9N_9/N_5N_5
GM109	23	/	23/23	6/23	17/23	N_9N_9/D_5N_5
GM105	27	/	27/27	6/27	21/27	N_9N_9/D_5N_5

Table S2. Oligonucleotides used for cloning of sgRNA or Cas9 and construction of vector for template preparing by in vitro transcription.

**Oligoes or Primers**	**Direction**	**Sequence(5' to 3')**
Cas 9	F	TAATACGACTCACTATAGGGAGAATGGACTATAAGGACCACGAC
Cas 9	R	GCGAGCTCTAGGAATTCTTAC
ASIP -sgRNA	CF	caccgTTTCCCTTCTGTCTCTATCG
ASIP -sgRNA	CR	aaacCGATAGAGACAGAAGGGAAAc
ASIP -sgRNA	TF	ttaatacgactcactataggTTTCCCTTCTGTCTCTATCG
ASIP -sgRNA	TR	AAAAGCACCGACTCGGTGCC
ASIP -F	F	CCAAGGAAACAAAGAAAGCAG
ASIP -R	R	AACCAAACAAGTTAAGGGACA

Footnotes: Cas9-F/R, primers used to amplify Cas9 gene as template for reverse transcription； *ASIP*-sgRNA-CF/CR: synthesized oligo nucleotides and complimentary strand used for annealing and generating sgRNA； *ASIP*-sgRNA-TF/TR: primers used to amplify template from plasmid for reverse transcription of sgRNA; *ASIP*-F/R： primers used to amplify targeting region for analysis of targeting events.
